# Retrospective Screening and Analysis of *mcr-1* and *bla*_NDM_ in Gram-Negative Bacteria in China, 2010–2019

**DOI:** 10.3389/fmicb.2020.00121

**Published:** 2020-02-11

**Authors:** Rong Fan, Chuchu Li, Ran Duan, Shuai Qin, Junrong Liang, Meng Xiao, Dongyue Lv, Huaiqi Jing, Xin Wang

**Affiliations:** ^1^State Key Laboratory of Infectious Disease Prevention and Control, Collaborative Innovation Center for Diagnosis and Treatment of Infectious Diseases – National Institute for Communicable Disease Control and Prevention, Chinese Center for Disease Control and Prevention, Beijing, China; ^2^Department of Acute Infectious Disease Control and Prevention, Jiangsu Provincial Center for Disease Control and Prevention, Nanjing, China

**Keywords:** MCR, NDM, polymyxin, carbapenem, Gram-negative

## Abstract

Currently, Gram-negative bacteria have developed multidrug and broad-spectrum drug resistance, and the numbers of species and strains carrying *mcr* or *bla*_NDM_ genes are increasing. In this study, *mcr-1* and *bla*_NDM_ distribution of 12,858 Gram-negative bacteria isolated from wildlife, patients, livestock, poultry and environment in 14 provinces of China from 2010 to 2019 and the antibiotics resistance in regard to polymyxins (polymyxin B and colistin) and carbapenems of positive strains were investigated. A total of 70 strains of 10 species carried the *mcr-1* gene, positive rates of patients, livestock and poultry, and environmental strains were 0.62% (36/5,828), 4.07% (29/712), 5.43% (5/92), respectively. Six strains of 3 species carrying the *bla*_NDM_ gene all came from patients 0.10% (6/5,828). Two new *mcr-1* gene variants (GenBank: MK965883, MK965884) were identified, one of which contains premature stop codon. The drug susceptibility results showed that all *mcr-1* carriers were sensitive to carbapenems, among which, 66 strains were resistant and 4 were sensitive to polymyxins. The strains with the *bla*_NDM_ gene had different degrees of resistance to carbapenems and were sensitive to polymyxins. The findings that species carrying *mcr-1* or *bla*_NDM_ genes were limited and mostly normal flora of opportunistic or low pathogenic organisms indicated that transfer of *mcr-1* and *bla*_NDM_ genes between bacteria was relatively limited in China. The none detection among wildlife compared with other sources supports the speculation that the emergence of and increase in polymyxins and carbapenem-resistant strains was mainly related to the selective pressure of antibiotics.

## Introduction

Bacterial resistance has been a global public health concern (Nolte, 2014; Alos, 2015). Currently, Gram-negative bacteria are developing multidrug resistance and broad-spectrum drug resistance, and the available antibiotics used in clinical treatment, agricultural and livestock production are gradually decreasing (Abdelraouf et al., 2017; Theuretzbacher, 2017). Polymyxins and carbapenems are among the antibiotics of last resort to treat Gram-negative bacteria infections. In 2009 and 2016, the superbug that carried the New Delhi metallo-beta-lactamase gene (*bla*_NDM–__1_) (Yong et al., 2009; Kumarasamy et al., 2010) and the *Escherichia. coli* that carried the colistin resistance gene (*mcr-1*) (Liu et al., 2016) were identified. Until now, 9 subtypes of *mcr* (Liu et al., 2016; Xavier et al., 2016; AbuOun et al., 2017; Borowiak et al., 2017; Carattoli et al., 2017; Yin et al., 2017; Wang X. et al., 2018; Yang et al., 2018; Carroll et al., 2019) and 21 subtypes of *bla*_NDM_ (Liu et al., 2018) have been published with papers. The discovery of these two types of resistance genes that can be horizontally transferred via plasmids has made researchers aware of the post-antibiotic era. Such plasmids usually carry other resistance genes, encoded for aminoglycosides and quinolones for instance (Carattoli, 2013; Rozwandowicz et al., 2018). The rapid horizontal spread of drug-resistant genes by plasmids is one of the reasons for the increasing number of multidrug resistant bacteria, however, the transfer range of bacteria species is unknown. Studies have confirmed that many countries and regions have isolated Gram-negative bacteria with *mcr-1* gene (Fernandes et al., 2016; Hadjadj et al., 2017; Liu et al., 2017a), the *bla*_NDM–__1_ gene (Wang et al., 2014; Michael et al., 2015; Zenati et al., 2016; Abderrahim et al., 2017; Madec et al., 2017; Riazzo et al., 2017; Yu et al., 2017), or both (Zheng et al., 2016; Liu et al., 2017b; Wang R. et al., 2018), from humans, the environment and animals. Whether isolates from antibiotics-free and depopulated areas, wildlife, carried *mcr* or *bla*_NDM_ genes is undiscovered.

To explore the transfer range of polymyxins and carbapenems resistance genes, 12,858 Gram-negative isolates of ≥118 species were retrospectively collected. Considering limited genes can be screened for the 12,858 isolates, only the *mcr-1* and *bla*_NDM_ genes were chosen, for their wide dissemination around the world and across species (López et al., 2019). To confirm the effect of antibiotics on the emergence of resistant strains, strains isolated from wildlife, patients, livestock, poultry, and environment from past 10 years were screened. Strains positive with either of the gene were confirmed by open reading frame (ORF) sequencing, and were tested for antimicrobial susceptibility.

## Materials and Methods

### Bacteria Isolation and Identification

We retrospectively collected 12,858 Gram-negative bacteria that were isolated from wildlife (6,226/12,858), patients (5,828/12,858), livestock and poultry (712/12,858), and environment (92/12,858) in 14 provinces (Anhui, Beijing, Gansu, Guangxi, Guizhou, Hainan, Hunan, Jiangxi, Ningxia, Qinghai, Sichuan, Tianjin, Yunnan, Zhejiang) of China from 2010 to 2019. The study was approved by the ethics review committee of the National Institute for Communicable Disease Control and Prevention, Chinese Center for Disease Control and Prevention. Informed consent was obtained from participants. All strains were identified using VITEK II Compact system (bioMérieux, France) or API 20E strips (bioMérieux, France). The *mcr-1* or *bla*_NDM_ gene positive strains were identified again by VITEK II Compact system (bioMérieux, France), the results of which were consistent with the original ones.

### Screening and ORF Sequencing of *mcr-1* or *bla*_NDM_ Positive Strains

DNA templates were extracted by using TIANamp Bacteria DNA Kit. Positive controls were used for PCR. All 12,858 Gram-negative bacteria were screened for *mcr-1* and *bla*_NDM_ gene by screening primers (Table 1), and ORF of positive strains were further amplified, cloned and sequenced. The *mcr-1* or *bla*_NDM_ positive strains were confirmed only if ORF sequences were obtained.

**TABLE 1:** The primers for screening and ORF amplification of the *mcr-1* and *bla*_NDM_ genes.

Primer	Sequence (5′ → 3′)	Product size (bp)	Annealing temperature (°C)
***mcr-1***			
MCR-1_CLR5-F	CGGTCAGTCCGTTTGTTC	309^a^	54
MCR-1_CLR5-R	CTTGGTCGGTCTGTAGGG		
***bla*_NDM_**			
NDM-1_17U-F	CAGCACACTTCCTATCTC	292^a^	54
NDM-1_17U-R	CCGCAACCATCCCCTCTT		
***mcr-1***			
*mcr-1* FL-F	AGAAGCACTGGGTGTAGAAT	2189^b^	54
*mcr-1* FL-R	GCCATGACAAGAGCGATA		
***mcr-1***			
FR-*mcr*-FL-F	CATCAATCAGTGGAGCG	2060^b^	54
FR-*mcr*-FL-R	CTCATCTCAGCAAGTAGG		
***mcr-1***			
DR-*mcr*-FL-F	GCAGTATAATTGCCGTAA	1841^b^	50
DR-*mcr*-FL-R	CTGACTGTGCTCAAGGGT		
***bla*_NDM_**			
21U-FL-F	TCGCATAAAACGCCTCTG	1007^b^	54
21U-FL-R	GAAACTGTCGCACCTCAT		

*^*a*^Screening, ^*b*^ORF amplification.*

### Antimicrobial Susceptibility Testing

The minimum inhibitory concentrations (MICs) of polymyxins (polymyxin B and colistin) and carbapenems of 70 strains with *mcr-1* and 6 strains with *bla*_NDM_ were determined by broth microdilution method in accordance with the Clinical and Laboratory Standards Institute (CLSI) guidelines (Clinical and Laboratory Standards Institute, 2019). Replication of sensitivity testing was conducted. Quality controls and breakpoints were in accordance with the European Committee on Antimicrobial Susceptibility Testing (2019) and the Clinical and Laboratory Standards Institute (2019) for polymyxins and carbapenems, respectively.

## Results

### Distribution of *mcr-1* or *bla*_NDM_ Positive Strains

A total of 70 strains with *mcr-1* and 6 strains with *bla*_NDM_ were confirmed. *mcr-1* positive strains were isolated each year since 2012. The positive rate from 2012 to 2019 was 0.31% (2/649), 0.09% (1/1,169), 0.63% (7/1,105), 0.36% (9/2,520), 0.64% (34/5,334), 0.28% (4/1,420), 2.12% (9/424), and 8.16% (4/49), respectively. The isolation rates of *bla*_NDM_ positive strains were 0.08% (2/2,520) in 2015, 0.06% (3/5,334) in 2016, and 0.24% (1/424) in 2018 (Table 2). Among the 70 strains with *mcr-1*, 36 were isolated from patients, with a positive rate of 0.62% (36/5,828) (Table 3). Thirty-five were isolated from diarrheal stool and 1 was from the sputum of an acute-pancreatitis patient. Twenty-nine strains were isolated from livestock and poultry specimens, with a positive rate of 4.07% (29/712), of which 24 isolates were from pig feces and 5 were from chicken feces. Five strains were isolated from environmental specimens, related to chicken slaughter, with a positive rate of 5.43% (5/92). Six *bla*_NDM_ positive strains were isolated from stool specimens of diarrhea patients, with a positive rate of 0.10% (6/5,828). None of the strains isolated from wild animal specimens were *mcr-1* or *bla*_NDM_ positive, which accounted for 48.42% (6,226/12,858) of all screened strains. These strains were isolated from rodents besides marmots (66.48%, 4,139/6,226), marmots (19.03%, 1,185/6,226), birds (6.10%, 380/6,226), plateau pika (4.34%, 270/6,226) and bats (4.05%, 252/6,226).

**TABLE 2:** The positive rate of strains with *mcr-1* or *bla*_NDM_ isolated in different years.

	No. *mcr-1* positive strains (%)	No. *bla*_NDM_ positive strain (%)
2010	None	None
2011	None	None
2012	2 (0.31)	None
2013	1 (0.09)	None
2014	7 (0.63)	None
2015	9 (0.36)	2 (0.08)
2016	34 (0.64)	3 (0.06)
2017	4 (0.28)	None
2018	9 (2.12)	1 (0.24)
2019	4 (8.16)	None
Total	70 (0.54)	6 (0.05)

*None: no strain positive for the *mcr-1* or *bla*_NDM_ gene.*

**TABLE 3:** The positive rate of strains with *mcr-1* or *bla*_NDM_ isolated from different sources.

	No. strains	No. *mcr-1* positive strains (%)	No. *bla*_NDM_ postive strains (%)
Wildlife	6,226	None	None
Livestock and poultry	712	29 (4.07)	None
Environment	92	5 (5.43)	None
Patients	5,828	36 (0.62)	6 (0.10)
Total	12,858	70 (0.54)	6 (0.05)

*None: no strain positive for the *mcr-1* or *bla*_NDM_ gene.*

**FIGURE 1:**
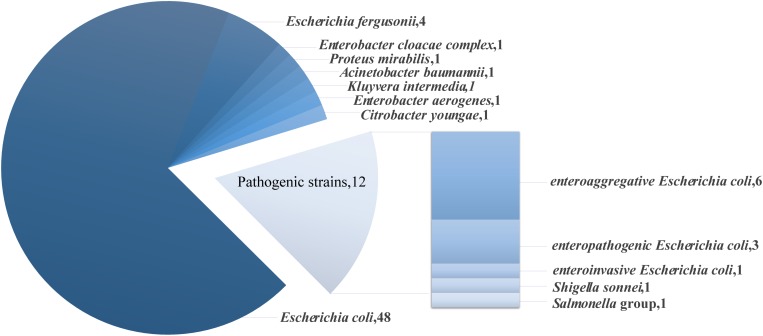
Species and number of *mcr-1* positive strains.

Of the *mcr-1* positive strains, 83% were opportunistic or low pathogenic organisms, including 48 strains of *Escherichia coli*, 4 strains of *Escherichia fergusonii*, 1 strain of *Enterobacter cloacae complex*, 1 strain of *Proteus mirabilis*, 1 strain of *Acinetobacter baumannii*, 1 strain of *Kluyvera intermedia*, 1 strain of *Enterobacter aerogenes* and 1 strain of *Citrobacter youngae* (Figure 1). The remaining 17% of the strains were pathogenic strains, including 6 strains of *enteroaggregative Escherichia coli* (EAEC), 3 strains of *enteropathogenic Escherichia coli* (EPEC), 1 strain of *enteroinvasive Escherichia coli* (EIEC), 1 strain of *Salmonella* group and 1 strain of *Shigella sonnei*. Strains carrying the *bla*_NDM_ gene that were low pathogenic or opportunistic organisms were 3 strains of *Escherichia coli*, 2 strains of *Klebsiella pneumoniae ssp. pneumoniae* and 1 strain of *Klebsiella oxytoca*.

### Sequence Analysis of *mcr-1* and *bla*_NDM_

**FIGURE 2:**
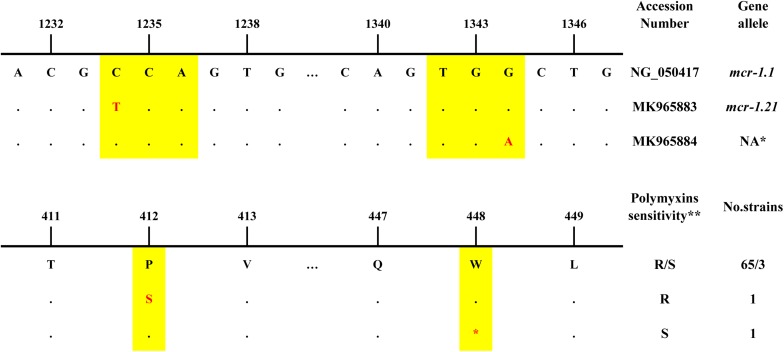
The nucleotide differences, amino acid differences and information of *mcr-1* positive strains in this study *NA: not assigned by NCBI. **R: resistant, S:sensitive.

The sequence alignment of the ORF showed that 68 of 70 *mcr-1* positive strains possessed an identical sequence compared to the reference sequence of the *mcr-1.1* gene, 1626 bp (NCBI Reference Sequence: KP347127, region: 22413-24038) (Figure 2). Among these strains, 65 were resistant while 3 were sensitive to polymyxins. The additional 2 *mcr-1* positive strains had a single base mutation compared to the reference sequence. One possessed *mcr-*1.21 (GenBank: MK965883), an *Escherichia coli* isolated from pig feces in Qinghai in 2016, which mutated at 1234 nt of reference sequence from C to T, resulting in a proline to serine change at the amino acid level. This strain shows resistance to polymyxins. The other strain possessed MK965884, an EAEC isolated from the stool of a diarrhea patient in Beijing in 2012. A base mutation site was located at 1344 nt of the reference sequence from G to A, resulting in a stop codon. The susceptibility results showed sensitivity to polymyxins.

Of 6 *bla*_NDM_ positive strains, 3 had identical sequences compared to the 813 bp reference sequence in the ORF of the *bla*_NDM–__1_ gene (NCBI Reference Sequence: FN396876 REGION: 2420-3232), 2 were identical to the *bla*_NDM–__3_ gene (NCBI Reference Sequence: JQ734687 REGION: 1-813), and 1 was identical to the 813 bp reference sequence in the ORF of the *bla*_NDM–__5_ gene (NCBI Reference Sequence: JN104597 REGION: 115-927).

### Antimicrobial Susceptibility Result

**FIGURE 3:**
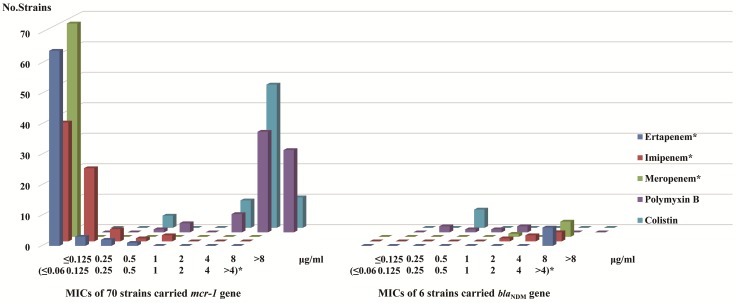
MICs distribution of carbapenem antibiotics and polymyxins in *mcr-1* and *bla*_NDM_ gene carriers MIC values (≤ 0.06 ∼>4 μg/ml)* in regard to ertapenem, imipenem, and meropenem. MIC values ≤0.125 ∼>8 μg/ml in regard to polymyxin B and colistin.

According to clinical breakpoint of carbapenem antibiotics, CLSI, all 70 strains carrying *mcr-1* showed sensitivity to ertapenem, imipenem, and meropenem. Among the 6 strains with *bla*_NDM_, 5 were resistant to ertapenem (MIC > 4 μg/ml), imipenem (3 strains MIC > 4 μg/ml, 2 strains MIC = 4 μg/ml) and meropenem (MIC > 4 μg/ml), while the other strain was resistant to ertapenem (MIC > 4 μg/ml) but intermediate to imipenem (MIC = 2 μg/ml) and meropenem (MIC = 2 μg/ml). According to clinical breakpoint of EUCAST, among the 70 strains with the *mcr-1* gene, 66 were resistant to polymyxins (polymyxin B: 6 strains MIC = 4 μg/ml, 33 strains MIC = 8 μg/ml, 27 strain MIC > 8 μg/ml. colistin: 9 strains MIC = 4 μg/ml, 47 strains MIC = 8 μg/ml, 10 strain MIC > 8 μg/ml) and the other four were sensitive to polymyxins (polymyxin B: 3 strains MIC = 1 μg/ml, 1 strain MIC = 0.5 μg/ml. colistin: 4 strains MIC = 0.5 μg/ml) (Table 4). Six strains with *bla*_NDM_ were sensitive to polymyxins (Figure 3). The sensitivity data for all strains referred to Supplementary Table S1.

**TABLE 4:** Strain information and MIC values of polymyxins sensitive strains.

	Bacteria	Host	Source	Year	*mcr-1* accession number	Polymyxin B MICs (μg/ml)	Colistin MICs (μg/ml)
QHXN2016-F75-2	*Escherichia coli*	Pig	Feces	2016	NG_050417	1	0.5
QHXN2016-F78-2	*Escherichia coli*	Pig	Feces	2016	NG_050417	1	0.5
GX1400471211	*Enterobacter aerogenes*	Diarrhea patient	Stool	2015	NG_050417	1	0.5
CY307223	*Escherichia coli*	Diarrhea patient	Stool	2012	MK965884	0.5	0.5

## Discussion

The increase in Gram-negative bacterial strains carrying the *mcr* or *bla*_NDM_ genes has led global health workers to reconsider infections and the treatment of bacteria infection (Kumarasamy et al., 2010). A total of 12,858 strains from ≥118 species of Gram-negative bacteria were screened, only to find 10 species carrying the *mcr-1* gene and 3 species carrying *bla*_NDM_ gene, indicating the species carrying these two genes and interspecific transfer of the two resistance genes were limited. The bacteria carrying these two resistance genes were mainly normal flora of opportunistic or low pathogenic organisms. Bacterial resistance is an ecological feature to maintain the lineage extension of the bacteria. The emergence of drug-resistant strains has a relationship with the widespread use of antibiotics (Liu et al., 2016). In fact, drug-resistant strains have existed for a long time (Haenni et al., 2016). Our study shows that the use of antibiotics may change the number of resistant strains, but it does not have an essential effect on the frequency of gene transfer. For example, metallo-β-lactamases itself is crucial in defining bacterial host specificity. The wider dissemination of NDM among different bacterial hosts, compared to VIM-2 and SPM-1, is mainly due to unique and singular features of this protein (López et al., 2019). In Gram-positive pathogens, the emergence of methicillin-resistant *Staphylococcus aureus* (MRSA) and vancomycin-resistant Staphylococcus aureus (VRSA) were alarming (Centers for Disease Control and Prevention, 2002). However, as of May 2015, only 14 VRSA infections have been reported in patients from the United States (Walters et al., 2015).

The total detection rate of this study was lower than other studies, mainly because 48.42% (6,226/12,858) of the strains were isolated from wildlife, while neither *mcr-1* nor *bla*_NDM_ genes was positive. Among the wildlife, marmots, plateau pika and bats live away from humans in this study. Rodents besides marmot (such as *Rattus flavipectus* and *Rattus norvegicus*) and birds mainly live between human surroundings and lands away from humans. The existence of antibiotic selective pressure away from humans is minimal, where no strain was found carrying *mcr-1* or *bla*_NDM_ gene. Among other sources, the highest detection rate of *mcr-1* was found in strains from the environment, which related to chicken slaughter (5.43%, 5/92), followed by livestock and poultry (4.07%, 29/712). Colistin has been used as a feed additive for livestock and poultry for growth promotion and disease prevention, especially for the treatment of gastrointestinal infections in livestock and poultry (Catry et al., 2015), which may cause widespread resistance of strains in livestock and poultry, further resulting in pollution of the environment, food, water and so on. The *mcr-1* gene was detected in bacteria isolated from imported chickens in Denmark (Hasman et al., 2015), food (Kuo et al., 2016; Yang et al., 2019) and wastewater (Zhao and Zong, 2016; Zhao et al., 2017) in China. In patients, the strains carrying the *mcr-1* gene (0.62%, 36/5,828) or *bla*_NDM_ gene (0.10%, 6/5,828) are possibly related to the use of polymyxins or carbapenems in hospitals and in-hospital transmission of drug-resistant strains. Therefore, it is possible that the increase in these two kinds of resistant strains is mainly due to the use of polymyxins or carbapenems antibiotics.

Different antibiotics usage may lead to various isolates selection. No resistant strains in this study co-harbored *mcr*-1 and *bla*_NDM–__1_ gene, while in other studies from China, such strains were seen (Zheng et al., 2016; Liu et al., 2017b; Wang R. et al., 2018). For instances, in patients, different antibiotics treatment were adopted according to different illness and severity. For livestock and poultry, antibiotics usage may also varied with breeding scales and farms. In addition to *mcr-1* and *bla*_NDM–__1_ gene, other genotype combination were also reported from China (Kong et al., 2017; Liu et al., 2017b; Chavda et al., 2018; Wang Q. et al., 2018; Wang R. et al., 2018). The *mcr* and *bla*_NDM_ genes co-harbored isolates from China are often *E. coli*., and *E. cloacae*, etc., occasionally. Further restrictions should be made on antibiotics usage for relevant occasions.

Compared with the ORF of the *mcr-1.1* gene, it was found that two strains have variants. One variant *mcr-*1.21 (GenBank: MK965883), changed from C to T at locus 1234 nt, which changed the codon from proline to serine but did not affect the polymyxin-resistant phenotype of the strain. The other variant (GenBank: MK965884) changed from G to A at locus 1344 nt, resulting in a codon change from tryptophan to stop codon. Strain of this variant was sensitive to polymyxins. It was speculated that the translation of this *mcr-1 gene* was terminated when containing premature stop codon and the functional *mcr-1* protein was not completely expressed, it truncated MCR family phosphoethanolamine – lipid A transferase. These two strains were isolated from pig and diarrhea patients, respectively, implying that mutations may be in response to antibiotic selection and certain environmental stresses. Some researchers have also detected variants of the *mcr-1* gene from bacteria isolated in environment or healthy individuals (Lu et al., 2017). In addition, three strains with the *mcr-1* gene were found to be sensitive to polymyxins, suggesting that the gene does not play a role in certain strains (Quan et al., 2017), which may be related to the metabolism of the strains. Another possible mechanism is the inhibition of *mcr-1* gene expression or the inactivation of phosphoethanolamine transferase it encodes (Liassine et al., 2016). In addition, multiple primers were designed to amplify the ORF of *mcr-1* positive strains, indirectly reflecting that the flanking regions of the ORF in this study may be variable. Variations did existed in the upstream of the ORF, compared with reference plasmid (KP347127): sequences amplified by the primer *mcr-1* FL showed T to C mutation at 22377nt. As to sequences amplified by primer FR-*mcr*-FL, shortly after the forward primer (KP347127:22091-22107) was a homologous fragments of ≥256 bp, which should be located at 20897-21152 of KP347127, prior to the location of forward primer.

Many genes are responsible and important for polymyxins or carbapenems resistance, e.g., *bla*_OXA_ (Evans and Amyes, 2014) and *bla*_KPC_ (Pitout et al., 2015). To focus on both mobile and widespread genes, only *mcr-1* and *bla*_NDM_ genes were screened for 12,858 strains, which is on the other hand, the limitation of the study. There may exist other important resistance mechanisms or genes in our strains. We would further resolve it using strains in this study and isolated afterward. The none detection of wildlife isolates from depopulated areas, in sharp contrast with positive findings of isolates from antibiotics-using areas, emphasized the importance of antibiotics management. On the other hand, the majority of strains collected from wildlife and patients is also the limitation of the study and should be considered when making conclusion.

The *mcr-1* and *bla*_NDM_ genes, mainly encoded by plasmids (Kumarasamy et al., 2010; Liu et al., 2016), have caused harm to livestock, poultry, humans and the environment, and therefore active measures should be taken against the bacteria. Our study have demonstrated that the transfer of *mcr-1* or *bla*_NDM_ genes between bacteria may be limited in China, however, the emergence of and increase in polymyxins and carbapenem-resistant strains was mainly related to the selective pressure of antibiotics. When using polymyxins and carbapenems antibiotics for disease prevention and control in the clinical setting, poultry, livestock and environment, strict management of usage is essential (Walsh and Wu, 2016; Al-Tawfiq et al., 2017), to prevent further intra- and interspecies dissemination of resistance genes and to control the spread of a broad-spectrum drug resistant or multidrug resistant bacteria.

## Data Availability Statement

The datasets generated for this study can be found in the https://www.ncbi.nlm.nih.gov/pathogens/isolates#/refgene/gene_family:(blaNDM), https://www.ncbi.nlm.nih.gov/pathogens/isolates#/refgene/gene_family:(mcr-1), MK965884.

## Author Contributions

XW and HJ contributed to the conception and design of the work. RF, CL, SQ, JL, MX, and DL performed the experiments. RF, CL, and RD drafted the manuscript. RF and RD performed the analysis and interpretation of the data. XW supervised the work. All authors read and approved the final version of the manuscript.

## Conflict of Interest

The authors declare that the research was conducted in the absence of any commercial or financial relationships that could be construed as a potential conflict of interest.
